# Maternal diet quality and nutrient intake in the gestational period: results from the delta healthy sprouts comparative impact trial

**DOI:** 10.1186/s40748-016-0036-7

**Published:** 2016-08-17

**Authors:** Lisa M. Tussing-Humphreys, Jessica L. Thomson, Melissa H. Goodman, Sarah Olender

**Affiliations:** 1 Department of Medicine and Cancer Center, University of Illinois at Chicago, 1747 W Roosevelt Road, #416, Chicago, IL 60618 USA; 2 United States Department of Agriculture, Agricultural Research Service, 141 Experiment Station Road, Stoneville, MS 38776 USA

**Keywords:** Maternal diet quality, Pregnancy, African American women

## Abstract

**Background:**

A woman’s diet while pregnant can play an important role in her reproductive health as well as the health of her unborn child. Diet quality and nutrient intake amongst pregnant women residing in the rural Lower Mississippi Delta (LMD) region of the United States is inadequate. The Delta Healthy Sprouts Project was designed to test the comparative impact of two home visiting programs on weight status, dietary intake, and health behaviors of women and their infants residing in the LMD region. This paper reports results pertaining to maternal diet quality and nutrient intake in the gestational period.

**Methods:**

The experimental arm (PATE) received monthly home visits beginning in the second trimester using the Parents as Teachers curriculum enhanced with a nutrition and lifestyle behavior curriculum. The control arm (PAT) received monthly home visits using the Parents as Teachers curriculum only. Maternal diet was assessed via 24-h dietary recall at gestational months (GM) 4 (baseline), 6, and 8. Diet quality was computed using the Healthy Eating Index-2010 (HEI-2010).

**Results:**

Gestational period retention rates for PAT and PATE arms were 77 % (33/43) and 67 % (26/39), respectively. Significant effects were not found for time, treatment, or time by treatment for the HEI-2010 total or component scores, macro- or micronutrient intake or percentage of women meeting recommended nutrient intakes.

**Conclusions:**

Perhaps due to low participant enrollment and higher than expected rates of drop out and noncompliance, we were not able to demonstrate that the enhanced nutrition and lifestyle curriculum (PATE) intervention had a significant effect on diet quality or nutrient intake during pregnancy in this cohort of rural, Southern, primarily African American women.

**Trial registration:**

clinicaltrials.gov, NCT01746394. Registered 5 December 2012.

## Background

A woman’s diet throughout pregnancy can play an important role in her reproductive health as well as the health of her unborn child [1]. Modest increases in energy intake in the 2^nd^ and 3^rd^ trimester and greater intake of several micronutrients including iron and folate throughout the gestational period are necessary to support a healthy pregnancy. Sub-optimal maternal nutrition is linked to adverse pregnancy outcomes including excessive maternal weight gain, the development of gestational complications including gestational diabetes mellitus, increased rates of preterm birth, infant growth restriction, and maternal and infant morbidity and mortality [2, 3].

Historically, research examining maternal diet has focused on the adequate intake of single nutrients during the gestational period [4, 5]. Although specific nutrients are indeed critical during pregnancy, nutritional research has shifted away from examining single nutrients and has begun to focus on examining the relationship between overall diet, specifically maternal diet quality, and maternal and fetal health [6]. This approach allows for the examination of total diet and the synergy of nutrients eaten together from various foodstuffs [7]. Relatively few studies have examined associations between maternal diet quality and pregnancy related outcomes [5]. However, there is some evidence that consuming a higher quality diet in the gestational period is associated with a reduction in maternal depressive symptoms [8], lower maternal fasting plasma glucose and reduced risk for gestational diabetes mellitus [9], and reduced risk for preeclampsia [10]. A recent summary of the limited but existing evidence suggests that a high-quality maternal diet consumed throughout the gestational period may reduce the risk for preterm birth, infant growth restriction, and fetal anomalies including neural tube defects [5]. Thus, interventions designed to optimize maternal diet during the gestational period have the potential to positively impact both maternal and fetal health.

One method to assess diet quality in pregnancy is to determine a woman’s adherence to the Dietary Guidelines for Americans (DGAs) [11] using the Healthy Eating Index (HEI) [12]. The HEI was designed by the United States Department of Agriculture, Center for Nutrition Policy and Promotion, to monitor diet quality in the United States (US) population and is appropriate for monitoring diet quality in at-risk low income sub-populations. The HEI ranges from 0 to 100 points with scores above 80 points indicative of a higher quality diet. In non-pregnant adults, a higher HEI score has been associated with lower total and cause-specific mortality [13] and lower prevalence of metabolic syndrome [14]. The HEI has been used successfully to assess diet quality in pregnancy [15]. However, Pick and colleagues noted that HEI failed to pick up inadequate micronutrient intake in a cohort of pregnant women. This finding suggests that when measuring diet quality in pregnancy, HEI may need to be modified to include key micronutrients (i.e., iron, folate, and calcium) [3, 16] or used in conjunction with more detailed nutrient intake data [17, 18].

The Lower Mississippi Delta (LMD) region of Mississippi is characterized by low education attainment and high rates of both poverty and chronic diseases including hypertension and obesity [19] and constitutes a majority African American population. Reproductive age women residing in this region suffer from some of the highest rates of adverse pregnancy-related outcomes in the US including maternal mortality, infant mortality, preterm birth, and low infant birth weight [20, 21]. Women of lower socioeconomic status, such as those residing in the LMD region, are at risk of being undernourished when they enter pregnancy due to poor diet and shorter intervals between pregnancies which can adversely impact a woman’s nutritional state both pre-conception and during pregnancy [22]. We recently reported that maternal diet quality and nutrient intake in the early 2^nd^ trimester amongst pregnant women residing in the LMD region is strikingly inadequate [23]. Overall, these women had very poor adherence to the 2010 DGAs based on HEI, and only a limited number of women met nutrient intake recommendations for fiber, sodium, calcium or choline. This was largely attributed to their low consumption of greens and beans, fruit, whole grains, and seafood and plant proteins – foods which have relatively high micronutrient and fiber, and low saturated fat and sodium contents. Given that poor maternal nutrition can affect not only the woman but her growing fetus, the diets of these women deserve considerable attention. Clearly, there is a need for interventions in the LMD region that are designed to help women achieve optimal nutrition through improved diet quality and nutrient intake throughout pregnancy.

The Delta Healthy Sprouts Project was designed to test the comparative impact of two maternal, infant, and early childhood home visiting programs on weight status, dietary intake, and health behaviors of women and their infants residing in the rural LMD region of Mississippi [24]. Results of the primary outcome, gestational weight gain, have been previously reported (Thomson, under review). This paper describes and compares the dietary intake outcomes of Delta Healthy Sprouts participants during the gestational period.

## Methods

### Design and recruitment

**Fig. 1:**
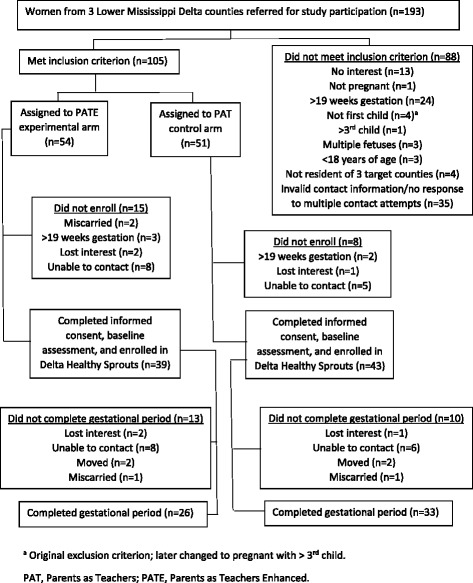
CONSORT diagram of recruitment, assignment, enrollment, and completion of gestational period for Delta Healthy Sprouts Project. ^a^ Original exclusion criterion; later changed to pregnant with > 3^rd^ child

This was a longitudinal analysis of the Delta Healthy Sprouts participants’ diet behaviors measured at the baseline [enrollment; gestational month (GM) 4] visit and two subsequent gestational (GM 6 and 8) visits. A comprehensive description of the Delta Healthy Sprouts Project has been published elsewhere [24]. Briefly, 82 pregnant women were enrolled in this on-going project conducted in three LMD counties. Inclusion criteria included female gender, at least 18 years of age, less than 19 weeks pregnant with first, second or third child, singleton pregnancy, and resident of Washington, Bolivar, or Humphreys County in Mississippi. Participant enrollment occurred on a rolling basis; hence baseline data were collected between March 2013 and December 2014. The target enrollment was 75 women in each of the two arms (control and experimental) of the project. The sample size of 150 women was based on the following assumptions: 20 % attrition rate, 37 % of control participants with GWG within the Institute of Medicine (IOM) recommendations, and a 22 % difference between treatment arms for GWG within recommendations [24]. However, recruitment was stopped by the study’s Principal Investigator prior to reaching these numbers due to unexpected difficulties recruiting pregnant women meeting study criteria. Recruitment was extended as long as possible, but fiscal issues eventually necessitated the closing of this period. Figure 1 illustrates the CONSORT diagram.

The project was approved by the Institutional Review Board of Delta State University, Cleveland MS (#12-024) and all participants gave written informed consent prior to any study engagement. Delta Healthy Sprouts is registered at clinicaltrials.gov (NCT01746394).

Delta Healthy Sprouts is evaluating the impact of the Parents as Teachers® (PAT) curriculum compared to a nutrition and physical activity enhanced PAT curriculum (PATE) on the primary outcomes of maternal gestational weight gain and postpartum weight control and childhood obesity prevention. Secondary outcomes pertain to maternal diet quality and nutrient intake during the gestational and postpartum periods, maternal physical activity, breast feeding and child feeding practices and other child-health related outcomes. Parents as Teachers is a nationally recognized, evidence based, home visiting program that seeks to increase parental knowledge of child development, improve parenting practices, provide early detection of developmental delays, prevent child abuse, and increase school readiness [25]. Participants were randomly assigned to one of two treatment arms [PAT control (*n* = 43) or PATE experimental (*n* = 39)] and are being followed for 18 months (6 months gestation and 12 months postnatal). At the baseline (GM 4) visit, demographic data and anthropometric measures were collected, 24-h dietary recalls were conducted, and physical activity and other questionnaires were administered.

### Intervention

The control arm of the intervention is based on the PAT curriculum that includes one-on-one home visits, developmental screenings, and a resource network for families. Home visitation is the key component of the PAT model where Parent Educators provide parents with research based information and activities. Materials are tailored to the age of the child and are responsive to parental information requests.

The experimental arm of the intervention builds upon the PAT curriculum by adding culturally tailored, maternal weight management and early childhood obesity prevention components. These features are based upon foundational elements from the Diabetes Prevention Program (DPP) and the Infant Feeding Activity and Nutrition Trial (InFANT). Elements based upon the DPP principles included a flexible, culturally sensitive, individualized educational curriculum taught on a one-to-one basis [26]. Elements taken from InFANT included anticipatory guidance and parenting support principles [27]. Anticipatory guidance involves providing practical, developmentally appropriate, child health information to parents in anticipation of significant physical, emotional, and psychological milestones [28]. Parenting support emphasizes children’s psychological and behavioral goals, logical and natural consequences, mutual respect, and encouragement techniques [28, 29].

For PATE, emphasis is placed on educating mothers about the ways in which they can facilitate the development of appropriate eating, physical activity, and other health behaviors in their children, including modeling these behaviors themselves. Intervention components of the PATE arm included appropriate weight gain during pregnancy and weight management after pregnancy, nutrition and physical activity in the gestational (mother) and postnatal (mother and infant) periods, breastfeeding, appropriate introduction of solid foods, and parental modeling of positive behaviors. Lessons included hands-on activities, instructional DVDs, and goal setting and reducing barriers for both diet and exercise. At each monthly visit in the gestational period, participants were given weight gain charts that contained reference ranges from the Institute of Medicine gestational weight gain recommendations. Participants’ current as well as past weight gain from previous visits was marked on these charts.

Both arms of the intervention were delivered in the home to women beginning in their early second trimester of pregnancy by trained community based Parent Educators. Parent Educators were African American college educated women residing in the target communities. They were trained to deliver the nutrition and physical activity lessons and to collect data from participants, including dietary intake. Home visits occurred monthly and were approximately 60–90 min in length for the PAT lessons, and approximately 90–120 min for the PATE lessons. Additional details regarding Parent Educator training, study methodology and lesson plan outlines have been published elsewhere [24].

### Measures

Anthropometric measures obtained on the participants at the baseline visit included height which was measured in duplicate using a portable stadiometer (model seca 217, seca, Birmingham, UK), and weight which was measured using a digital scale (model SR241, SR Instruments, Tonawanda, NY). Both measures were performed without shoes or heavy clothing. Pre-pregnancy weight was self-reported. Body mass index (BMI) was calculated as weight (kg) divided by height (m) squared where height was averaged if the two measurements differed. Weight also was measured at each of the five subsequent gestational visits. Weight gain is examined in another paper (Thomson, under review).

Dietary data were collected from the participants at the baseline, GM 6, and GM 8 visits via multiple pass 24-h dietary recall using Nutrition Data System for Research (NDSR) software. NDSR is a Windows-based dietary analysis program that allows for the calculation of nutrients per ingredient, food, meal, and day in report and analysis formats [30]. The software also includes a dietary supplement assessment module so that nutrient intake from supplemental sources can be captured and quantified [31]. Using the dietary data collected with NDSR, participants’ diet quality was calculated using the Healthy Eating Index-2010 (HEI-2010) [12], which measures adherence to the 2010 DGAs. The HEI-2010 includes 12 components that are summed to create a total score ranging from 0 to 100 points. The 12 components include: total vegetables, greens and beans, total fruit, whole fruit, whole grains, dairy, total protein foods, seafood and plant protein foods, fatty acids, refined grains, sodium, and empty calories. For each component, higher scores reflect greater adherence to the 2010 DGAs. An HEI score greater than 80 is representative of a good quality diet; 51 – 80 is consistent with a diet that needs improvement in regard to quality; and a score less than 51 represents a poor quality diet [32]. Additionally, intakes of specific nutrients of interest were determined using the dietary data collected. These nutrients (total and saturated fat, cholesterol, sodium, calcium, fiber, folate, iron, vitamins C and D, and choline) were chosen based on their importance to an overall healthy diet and increased nutrient needs during pregnancy [33–37].

Participants also provided information regarding demographic characteristics (e.g., age, marital status, household size, education, employment, household income, insurance, prenatal care), health history, and current health conditions. Details regarding other measures and questionnaire data that were collected using validated tools, but are not relevant to the current paper, have been published elsewhere [24]. All measures, dietary recalls, and questionnaires were collected or administered by trained research staff (Parent Educators) using laptop computers loaded with relevant software (i.e., NDSR and Snap Surveys).

### Statistical analyses

Statistical analyses were performed using SAS® software, version 9.4 (SAS Institute Inc., Cary, NC). Descriptive statistics, including means, standard deviations, frequencies, and percentages, were used to summarize participants’ demographic characteristics, anthropometric measures, nutrient intake, and diet quality.

Chi square tests of association or Fisher’s exact tests (categorical measures) and two sample t tests (continuous measures) were used to assess differences between PAT and PATE participants’ baseline characteristics and between gestational period study completers’ and non-completers’ baseline characteristics. Study completers were defined as participants who had their GM 9 visit or those who had at least two visits in the gestational period and their post-natal 1 visit. The second definition was used because a substantial proportion of PAT and PATE participants (36 and 42 %) who had their post-natal 1 visit, missed their GM 9 visit due to the early birth of their infant.

Compliance with diet recommendations was determined based on current guidelines set by professional organizations. Participants were classified as compliant with dietary guidelines if they met minimum daily intake for fiber (28 g), calcium (1300 mg for women 18 years of age and 1000 mg for women ≥ 19 years of age), folate (600 mcg), iron (27 mg), vitamin C (80 mg for women 18 years of age and 85 mg for women ≥ 19 years of age), vitamin D (5 mcg), and choline (450 mg) or did not exceed the maximum daily intake for total dietary fat (35 % of total energy), saturated fat (10 % of total energy), cholesterol (300 mg), and sodium (1500 mg for African American women).

Generalized linear mixed models, using maximum likelihood estimation, were used to test for significant treatment, time, and treatment by time (interaction) effects on diet outcomes. Maximum likelihood estimation is an approach for handling missing data in repeated measures. Treatment (PAT vs. PATE) was modeled as a fixed effect and time (GM 4, GM 6, and GM 8 visits) was modeled as a repeated measure using a first-order autoregressive covariance matrix structure. If overdispersion was present, then a multiplicative overdispersion parameter was added to the model. Least squares means with 95 % confidence intervals were computed using these models. Distributions of the diet outcome variables were checked for approximate normality based on both goodness of fit tests (Cramer-von Mises and Anderson-Darling) and visual inspection. If the distribution passed the goodness of fit tests or failed the tests but appeared sufficiently normal for the underlying assumptions of normality to be reasonable for practical purposes of analysis, then the diet outcome (HEI total and component scores; and total fat, sodium, folate, and vitamin D intakes) was modeled using a Gaussian distribution with an identity link function. The remaining variables were checked for approximate gamma, inverse normal, and log normal distributions, again based on goodness of fit tests and visual inspection. Saturated fat and calcium intakes were modeled using a gamma distribution with a log link function. The remaining five diet outcome variables (cholesterol, dietary fiber, iron, vitamin C, and choline) were modeled using quantile regression on the median to test for significant treatment, time and treatment by time effects. Treatment and time were modeled as fixed effects and 95 % confidence intervals for the medians were computed using Markov chain marginal bootstrap resampling. Generalized linear mixed models also were used to test for significant treatment, time, and treatment by time effects on the proportions of participants who met recommended nutrient intakes using a binomial distribution with a logit link function. The significance level of the tests was set at 0.05.

## Results

Study retention rates during the gestational period for the PAT and PATE treatment arms were 77 % (33/43) and 67 % (26/39), respectively, and did not differ between arms (*p* = 0.310). Compliance rates for the GM 6 and GM 8 visits were 88 and 84 %, respectively, for PAT participants, and 67 and 51 %, respectively, for PATE participants. Compliance rates for both visits were significantly lower in the PATE arm (*p* = 0.018 and 0.002, respectively). One PAT participant did not provide dietary intake data at the GM 4 (baseline) visit. One PATE participant who missed her GM 6 visit provided dietary intake data during her GM 7 visit. Likewise, two PATE participants who missed their GM 8 visit provided dietary intake data during their GM 9 visit.

Table 1 presents comparisons between treatment arms for baseline characteristics. The majority of both PAT and PATE participants were African American (95 and 97 %), single (91 and 95 %), receiving Medicaid (93 and 90 %), overweight/obese prior to pregnancy (63 and 72 %), and started their prenatal care in their second month of pregnancy (56 % for both). Additionally, PAT and PATE participants were young (mean age = 23 ± 4.6 years and 23 ± 4.7 years) and early in their second trimester of pregnancy (mean gestational age = 17 ± 1.9 weeks and 18 ± 2.4 weeks). There were no significant differences between PAT and PATE participants at baseline. However, women that were retained throughout the gestational period were significantly more likely to have access to a motor vehicle as compared to those not retained (95 % vs. 78 %, *p* = 0.036; data not shown in the table).

**Table 1:** Delta Healthy Sprouts participant baseline (early second trimester of pregnancy) demographic characteristics by treatment arm

	PAT (*n* = 43)		PATE (*n* = 39)		
Characteristic	n	%	n	%	P
Race					1.000
African American	41	95.3	38	97.4	
White	2	4.7	1	2.6	
Marital status					0.678
Single^a^	39	90.7	37	94.9	
Married	4	9.3	2	5.1	
Education level					0.684
9th–11th grade	7	16.3	7	17.9	
High school/GED	15	34.9	12	30.8	
Some college/technical	17	39.5	13	33.3	
College degree	4	9.3	7	17.9	
Employment status					0.468
Full time/part-time	13	30.2	16	41.0	
Unemployed (looking)	21	48.8	14	35.9	
Homemaker/student	9	20.9	9	23.1	
Household monthly income^b^					0.284
< $500	10	23.3	5	12.8	
$500-$1000	10	23.3	9	23.1	
$1001–$1500	4	9.3	12	30.8	
$1501–$2000	7	16.3	5	12.8	
$2001–$4000	6	14.0	3	7.7	
Don’t know/refused	6	14.0	5	12.8	
Smoker in household	12	27.9	12	30.8	0.776
Smoker^c^					0.112
Current	3	7.0	1	2.6	
Stopped before pregnancy	1	2.3	0	0.0	
Stopped after became pregnant	2	4.7	0	0.0	
No	37	86.0	38	97.4	
Medicaid health insurance	40	93.0	35	89.7	0.703
Receiving SNAP	35	81.4	27	69.2	0.200
Receiving WIC	38	88.4	31	79.5	0.271
Own/access to vehicle	39	90.7	35	89.7	1.000
Receiving prenatal care	43	100.0	39	100.0	
Pre-pregnancy weight class^d^					0.386
Underweight (BMI < 18.5)	4	9.3	3	7.7	
Healthy weight (18.5 ≤ BMI < 25)	12	27.9	8	20.5	
Overweight (25 ≤ BMI < 30)	9	20.9	10	25.6	
Obese (BMI ≥ 30)	18	41.9	18	46.2	
	Mean	SD	Mean	SD	P
Age (years)	23.3	4.58	22.7	4.69	0.537
Household size	3.8	1.62	4.1	1.78	0.406
Gestational age^e^	17.4	1.85	17.7	2.43	0.533

*PAT* Parents as Teachers control treatment; *PATE* Parents as Teachers, Enhanced experimental treatment; *SNAP* Supplemental Nutrition Assistance Program; *WIC* SNAP for Women, Infants, and Children; *BMI* body mass index

^a^Includes 1 participant who indicated she is divorced

^b^Comparison = <$500–$1500 vs. $1501–$4000; don’t know/refused excluded

^c^Comparison = no vs. all other responses

^e^Based on self-reported pre-pregnancy weight; comparison = underweight or healthy weight vs. overweight or obese

^e^Based on reported due date; enrollment data collected late for 3 participants

### Diet quality and nutrient intake outcomes

Table 2 presents the results of the diet quality analyses by treatment arm and gestational visit. HEI-2010 total diet quality mean scores for PAT participants at GM 4, GM 6, and GM 8 were 41, 41, and 43 points, respectively; corresponding scores for PATE participants were 46, 44, and 45 points, respectively, reflecting overall poor diet quality for all participants at all gestational time points. In particular, HEI-2010 component mean scores were low for greens and beans, whole grains, seafood and plant protein foods, and sodium for both treatment arms. Significant treatment, time and interaction effects were not found for the HEI-2010 total or component scores.

**Table 2:** Diet quality scores for Delta Healthy Sprouts participants in the gestational period by treatment arm and visit (time)

		GM 4 (*n* = 43 and 38)^a^			GM 6 (*n* = 39 and 25)			GM 8 (*n* = 36 and 22)^b^			P		
HEI-2010 Component	Arm	LSM	95 % CI		LSM	95 % CI^c^		LSM	95 % CI^c^		Time	Arm	Int
Total vegetables	PAT	2.3	1.8	2.7	1.8	1.3	2.2	2.1	1.6	2.5	0.877	0.237	0.367
(Range 0-5)	PATE	1.7	1.3	2.2	1.9	1.4	2.5	1.8	1.2	2.4			
Greens & beans	PAT	0.6	0.1	1.0	0.8	0.3	1.3	0.5	0.0	1.0	0.243	0.820	0.797
(Range 0-5)	PATE	0.6	0.1	1.1	0.8	0.2	1.5	0.2	0.0	0.9			
Total fruit	PAT	1.3	0.7	1.9	2.2	1.5	2.8	1.5	0.8	2.1	0.713	0.860	0.086
(Range 0–5)	PATE	1.8	1.1	2.4	1.4	0.6	2.2	1.9	1.1	2.8			
Whole fruit	PAT	1.1	0.4	1.7	1.8	1.1	2.4	1.2	0.5	1.8	0.248	0.214	0.539
(Range 0–5)	PATE	1.6	0.9	2.2	1.8	1.0	2.6	1.9	1.1	2.8			
Whole grains	PAT	2.4	1.4	3.4	1.8	0.7	2.8	2.1	1.1	3.2	0.818	0.388	0.375
(Range 0–10)	PATE	2.5	1.4	3.5	3.1	1.8	4.4	2.1	0.7	3.5			
Dairy	PAT	4.4	3.5	5.2	3.6	2.7	4.5	4.0	3.1	4.9	0.311	0.086	0.543
(Range 0–10)	PATE	3.7	2.7	4.6	3.4	2.3	4.5	2.7	1.5	3.9			
Total protein foods	PAT	4.4	4.0	4.8	4.3	3.9	4.7	4.2	3.8	4.6	0.960	0.561	0.548
(Range 0–5)	PATE	4.3	3.9	4.7	4.5	4.0	4.9	4.6	4.0	5.1			
Seafood & plant proteins	PAT	0.7	0.1	1.3	1.0	0.4	1.6	0.8	0.2	1.4	0.960	0.813	0.362
(Range 0–5)	PATE	1.2	0.6	1.8	0.7	0.0	1.4	0.9	0.1	1.7			
Fatty acids	PAT	5.2	4.1	6.2	5.2	4.2	6.3	6.4	5.2	7.5	0.397	0.060	0.576
(Range 0–10)	PATE	6.8	5.7	7.9	6.3	5.0	7.7	6.8	5.3	8.2			
Sodium	PAT	2.4	1.4	3.3	2.9	2.0	3.9	2.9	1.9	3.9	0.780	0.118	0.653
(Range 0–10)	PATE	3.5	2.5	4.5	3.1	1.9	4.4	3.7	2.4	5.0			
Refined grains	PAT	5.8	4.8	6.9	4.5	3.4	5.6	5.3	4.2	6.5	0.054	0.104	0.554
(Range 0–10)	PATE	6.2	5.1	7.3	5.0	3.6	6.4	7.0	5.5	8.5			
Empty calories	PAT	10.5	8.8	12.2	10.7	9.0	12.5	12.2	10.4	14.1	0.759	0.450	0.519
(Range 0–20)	PATE	11.9	10.1	13.7	11.9	9.7	14.1	11.5	9.2	13.9			
Total	PAT	40.9	37.1	44.8	40.6	36.5	44.6	43.2	39.0	47.5	0.696	0.100	0.813
(Range 0–100)	PATE	45.6	41.5	49.7	43.9	38.9	49.0	45.1	39.7	50.5			

*GM* gestational month; *HEI-2010* Healthy Eating Index-2010; *LSM* least squares mean; *CI* confidence interval; *Int* interaction (time x arm); *PAT* Parents as Teachers control treatment; *PATE* Parents as Teachers Enhanced experimental treatment

^a^Missed dietary recall for 1 PATE participant

^b^Excluded dietary recall for 1 PATE participant (in hospital and consumed ice only)

^c^Negative lower limits are not feasible and were changed to 0

Table 3 presents the results of the nutrient intake analyses by treatment arm and gestational visit. Significant treatment, time and interaction effects were not found for any of the nutrients modeled. However, there was a clinically meaningful reduction in the percentage of total energy from saturated fat in the PATE arm between GM 4 and GM 8 (10.6 to 9.6 %). An unexpected but clinically meaningful reduction in mean calcium intake in the PATE arm between GM 4 and GM 8 (861 mg to 792 mg) also was observed.

**Table 3:** Nutrient intakes for Delta Healthy Sprouts participants in the gestational period by treatment arm and visit (time)

		GM 4 (*n* = 43 and 38)^a^			GM 6 (*n* = 39 and 25)			GM 8 (*n* = 36 and 22)^b^			P		
Nutrient	Arm	LSM	95 % CI		LSM	95 % CI		LSM	95 % CI		Time	Arm	Int
Total fat (% energy)	PAT	35.7	33.4	38.0	32.8	30.5	35.2	34.6	32.1	37.1	0.175	0.990	0.233
RDI ≤ 35 %	PATE	35.6	33.2	38.1	34.9	32.0	37.8	32.7	29.5	35.8			
Saturated fat (% energy)	PAT	11.4	10.4	12.5	10.7	9.7	11.7	10.5	9.5	11.6	0.204	0.178	0.780
RDI ≤ 10 %	PATE	10.6	9.6	11.6	10.4	9.3	11.7	9.6	8.5	10.8			
Cholesterol (mg)^c^	PAT	203	141	266	193	130	256	165	102	227	0.211	0.151	0.143
RDI ≤ 300 mg	PATE	179	116	242	248	185	311	228	166	291			
Fiber (g)^c^	PAT	10.7	8.4	13.1	11.7	9.4	14.0	11.3	9.0	13.6	0.881	0.607	0.869
RDI ≥ 28 g	PATE	10.2	7.9	12.5	11.3	9.0	13.6	10.4	8.1	12.7			
Sodium (mg)	PAT	3770	3332	4208	3839	3379	4298	3725	3246	4203	0.700	0.334	0.900
RDI ≤ 1500 mg	PATE	3624	3158	4090	3718	3144	4293	3378	2766	3990			
Calcium (mg)^d^	PAT	960	842	1094	939	818	1078	966	837	1115	0.305	0.281	0.151
RDI = 1000 mg	PATE	861	749	990	1015	855	1205	792	659	951			
Folate (mcg)	PAT	1246	1071	1421	1275	1092	1459	1252	1061	1444	0.904	0.278	0.990
RDI = 600 mcg	PATE	1142	956	1329	1200	970	1429	1157	912	1401			
Iron (mg)^c^	PAT	41.2	36.3	46.2	41.6	36.7	46.5	41.9	37.0	46.9	0.710	0.555	0.917
RDI = 27 mg	PATE	39.2	34.2	44.1	43.0	38.1	47.9	40.3	35.4	45.2			
Vitamin C (mg)^c,e^	PAT	162	117	207	197	152	241	183	139	228	0.690	0.471	0.947
RDI = 85 mg	PATE	153	109	198	179	134	224	163	118	208			
Vitamin D (mcg)	PAT	12.4	10.7	14.2	11.6	9.8	13.4	12.3	10.5	14.2	0.907	0.380	0.559
RDI = 5 mcg	PATE	10.9	9.1	12.8	11.3	9.2	13.4	11.0	8.8	13.2			
Choline (mg)^c^	PAT	240	181	299	248	189	307	206	147	265	0.745	0.392	0.403
RDI = 450 mg	PATE	223	164	282	280	221	339	239	180	298			

*GM* gestational month; *HEI-2010* Healthy Eating Index-2010; *LSM* least squares mean; *CI* confidence interval; *Int* interaction (time x arm); *PAT* Parents as Teachers control treatment; *PATE* Parents as Teachers Enhanced experimental treatment

^a^Missed dietary recall for 1 PATE participant

^b^Excluded dietary recall for 1 PATE participant (in hospital and consumed ice only)

^c^Values are medians and associated 95 % confidence intervals from quantile regression

^d^RDI = 1300 mg for participants 18 years of age

^e^RDI = 80 mg for participants 18 years of age

Table 4 presents the results of the proportions of participants meeting nutrient intake recommendations by treatment arm and gestational visit. At baseline < 20 % of women met intake recommendations for dietary fiber, sodium, or choline. Whereas, more than half of the women met intake recommendations for cholesterol, folate, iron, vitamin C, and vitamin D at baseline. Significant treatment, time and interaction effects were not found for any of the nutrients modeled. However, clinically meaningful increases in percentages of participants meeting recommendations for saturated fat intake between GM 4 and GM 8 were observed (PAT 30 to 42 %; PATE 47 to 64 %). Likewise, clinically meaningful increases in percentages of PAT participants meeting recommendations for cholesterol and vitamin C intakes between GM 4 and GM 8 were observed (65 to 84 % and 86 to 97 %, respectively). An unanticipated albeit clinically meaningful decrease in percentage of PATE participants meeting recommendations for calcium intake between GM 4 and GM 8 also was observed (29 to 13 %).

**Table 4:** Percentages of Delta Healthy Sprouts participants meeting recommended nutrient intakes in the gestational period by treatment arm and visit (time)

		GM 4 (*n* = 43 and 38)^a^			GM 6 (*n* = 39 and 25)			GM 8 (*n* = 36 and 22)^b^			P		
Nutrient^c^	Arm	LSM	95 % CI		LSM	95 % CI		LSM	95 % CI		Time	Arm	Int
Total fat (% energy)	PAT	48.8	34.1	63.8	53.9	38.0	69.0	49.7	33.6	65.9	0.650	0.467	0.684
RDA ≤ 35 %	PATE	50.0	34.2	65.8	55.4	35.8	73.6	64.5	42.6	81.6			
Saturated fat (% energy)	PAT	30.2	18.2	45.8	43.8	29.0	59.9	41.6	26.5	58.4	0.285	0.063	0.570
RDA ≤ 10 %	PATE	47.4	31.9	63.4	48.2	29.5	67.5	63.5	41.7	80.9			
Cholesterol (mg)	PAT	65.1	49.5	78.1	64.3	47.9	77.9	83.7	67.5	92.7	0.414	0.607	0.304
RDA ≤ 300 mg	PATE	68.4	51.8	81.4	69.1	48.5	84.2	67.1	45.1	83.6			
Fiber (g)	PAT	0.0	NC	NC	0.0	NC	NC	0.0	NC	NC	NC	NC	NC
RDA ≥ 28 g	PATE	7.9	NC	NC	0.0	NC	NC	9.1	NC	NC			
Sodium (mg)	PAT	0.0	NC	NC	2.6	NC	NC	5.6	NC	NC	NC	NC	NC
RDA ≤ 1500 mg	PATE	15.8	NC	NC	0.0	NC	NC	0.0	NC	NC			
Calcium (mg)^d^	PAT	41.9	27.9	57.3	40.7	26.3	57.0	44.3	28.8	61.0	0.245	0.054	0.130
RDA = 1000 mg	PATE	28.9	16.5	45.6	41.8	24.2	61.8	13.4	4.2	35.2			
Folate (mcg)	PAT	90.7	77.4	96.5	91.4	77.6	97.0	88.7	73.8	95.6	0.929	0.486	0.871
RDA = 600 mcg	PATE	86.8	71.8	94.5	85.1	66.9	94.1	85.7	65.5	95.0			
Iron (mg)	PAT	90.7	77.4	96.5	91.1	77.5	96.8	88.4	73.8	95.4	0.975	0.260	0.486
RDA = 27 mg	PATE	81.6	65.8	91.1	79.2	61.4	90.2	84.9	65.7	94.3			
Vitamin C (mg)^e^	PAT	86.0	71.8	93.7	89.4	74.9	96.0	97.3	82.3	99.6	0.267	0.184	0.495
RDA = 85 mg	PATE	84.2	68.6	92.9	81.4	61.6	92.3	87.0	65.6	95.9			
Vitamin D (mcg)	PAT	83.7	69.3	92.2	85.0	70.2	93.2	82.6	66.8	91.8	0.949	0.699	0.790
RDA = 5 mcg	PATE	78.9	62.9	89.3	79.9	61.4	90.9	83.4	63.2	93.6			
Choline (mg)	PAT	14.0	6.2	28.3	15.7	7.2	31.1	8.1	2.5	23.2	0.525	0.992	0.349
RDA = 450 mg	PATE	18.4	8.8	34.5	7.5	1.8	26.7	13.0	4.0	34.7			

*GM* gestational month; *HEI-2010* Healthy Eating Index-2010; *LSM* least squares mean; *CI*, confidence interval; *Int* interaction (time x arm); *PAT* Parents as Teachers control treatment; *PATE* Parents as Teachers Enhanced experimental treatment; *NC*, not computed since model failed to converge due to time points with zero and close to zero percentages

^a^Missed dietary recall for 1 PATE participant

^b^Excluded dietary recall for 1 PATE participant (in hospital and consumed ice only)

^c^Based on food and supplements combined

^d^RDA = 1300 mg for participants 18 years of age

^e^RDA = 80 mg for participants 18 years of age

## Discussion

In this paper, we describe and compare the gestational dietary intake outcomes of rural, Southern, primarily African American pregnant women participating in the Delta Healthy Sprouts randomized, comparative impact trial. Overall, our results suggest that the PAT curriculum and the more complex PATE intervention, were not effective at improving maternal diet quality or the adequacy of intake for several nutrients including dietary fiber, calcium and choline during the gestational period.

Other lifestyle interventions conducted in the gestational period have been successful at improving maternal diet quality and nutrient intake. The UPBEAT trial [38], conducted in the United Kingdom with a largely Caucasian population, was effective at improving diet quality in pregnant women with obesity. This one-on-one intervention was delivered in an antenatal clinic once weekly for 8 weeks with a health educator beginning between 16 and 19 weeks gestation. The intervention was successful at reducing maternal glycemic load and total and saturated fat intake and increasing dietary fiber. The LIMIT trial [18], a randomized prenatal dietary and lifestyle intervention conducted in a cohort of pregnant overweight and obese, predominately Caucasian, high SES, Australian women, was effective at improving maternal diet quality, measured by HEI, and intake of dietary fiber and saturated fat compared to standard care [18]. This intervention included six sessions implemented on an individual basis both in person and over the phone by a registered dietitian and trained research assistants. While our one-on-one intervention was implemented monthly between GM 4 and 9 in the participant’s home by a trained lay parent educator, only three of the five lessons (GM 5, 6 and 7) were delivered prior to the final gestational diet intake assessment at GM 8. It may be that the dose of our intervention was too low to foster or the timing of our final diet intake was not optimal for detecting positive dietary changes. Lifestyle changes, including dietary changes, involve the adoption of new positive behaviors [39] and research suggests that lifestyle habits can take anywhere from 66 to 254 days to form [40]. Thus, the short duration of our gestational intervention may not have allowed for the time needed to develop and adopt positive dietary behaviors [39]. Given that our intervention spans into the postpartum period, future analyses will assess the adoption of positive diet behaviors in this cohort of women over a longer period of time.

The UPBEAT [38] and LIMIT [18] prenatal lifestyle intervention trials focused their messaging solely on maternal gestational weight gain, diet, and lifestyle behaviors. The Delta Healthy Sprouts Project was built on the framework of the evidence-based PAT curriculum which covers topics related to parental knowledge of child development, positive parenting practices, early detection of developmental delays, preventing child abuse, and school readiness. Thus, combining the PATE diet and lifestyle curriculum with the existing PAT program may have diluted the diet and lifestyle messaging. Further, some studies suggest that multi-component dietary advice such as USDA MyPlate, which was used as the framework for the PATE dietary messaging, can be overwhelming to some individuals due to the knowledge required to comprehend the complex recommendations [41]. The considerable and diverse number of messages provided in the PATE curriculum may have overwhelmed the women randomized to this arm of the study. The higher attrition and lower compliance with home visits during the gestational period, despite no other notable participant characteristic differences, suggests some level of displeasure with the PATE lessons. The standard PAT curriculum provides some simple maternal dietary advice including importance of taking prenatal vitamins, safe and healthy fish options during pregnancy, and consuming more calories during the 2^nd^ and 3^rd^ trimester. The three point increase in total HEI-2010 score and improvements in saturated fat, cholesterol and vitamin C intake in the PAT arm suggests that simple nutrition messaging, when presented in the context of a more global maternal and child health curriculum, may promote positive maternal dietary behaviors in the gestational period. Further research is needed to better understand the impact of simple vs. complex nutrition messaging on maternal diet in rural, disadvantaged pregnant women.

Women are bombarded with information during pregnancy [39]. All of the women in our study were receiving prenatal care at baseline and more than 80 % of our participants were enrolled in the Women Infants and Children (WIC) supplemental nutrition program. Further, 88 and 83 %, respectively, of our PAT and PATE participants indicated they had received advice about healthy eating from their prenatal care provider. Thus, it is probable that participants were overwhelmed by a vast amount of health and lifestyle-related information and possible that our diet advice conflicted with advice received from their clinician or WIC counselor. Research suggests that pregnant women tend to place the most importance on recommendations made by their doctor/clinical provider [42, 43]. Whereas women tend to be somewhat dismissive of health recommendations that were not addressed by their health care provider. Therefore, women in the PATE arm may have been less likely to follow our diet recommendations if similar advice was not also encouraged by their provider [39]. Future interventions should consider involving obstetrical healthcare providers to allow for consistent messaging around gestational lifestyle behaviors.

Our study was implemented in the largely rural, socioeconomically disadvantaged LMD region. Studies suggest that socioeconomic status (SES) is positively associated with diet quality [44]. There are several hypothesized explanations for this relationship. Research suggests that low SES individuals may experience greater reward from food given their limited access to non-food related rewards due to cost or access [45]. Further, low SES neighborhoods and regions, like the LMD, have fewer large supermarkets and more convenience stores and fast-food outlets that offer relatively low cost, nutrient poor, energy dense foods [46]. Research has shown that living in low SES areas is associated with less consumption of fruits and vegetables and a tendency to select foods that are more readily accessible [47, 48]. Epstein and colleagues [45] suggest that even when access to healthier food is improved through participation in food assistance programs, low SES individuals will continue to choose foods of low nutritive value that provide greater food reinforcement. Epstein hypothesizes that reducing access to low quality foods through policy-related changes and increasing the availability of low-cost, non-food reinforcement, may encourage the selection of healthier food choices in low SES populations.

Several strengths of the Delta Healthy Sprouts Project deserve consideration. Participants were visited in their homes, thus alleviating the burden of travel for these rural residents. Although the study’s sample size is relatively small, no other studies were found which focused on the diet, including nutrient intake and diet quality, of this population of pregnant women. Dietary intake was measured three times in the gestational period (vs. the more typical pre/post measurement schedule). Despite the strengths of this study, there are some limitations that bear mentioning. The potential for socially desirable responses for survey questions and dietary recalls cannot be discounted. To reduce this bias potential, parent educators were trained to not ask leading questions and to maintain neutral facial expressions, particularly when conducting dietary recalls. Data collection was not blinded and therefore a potential source of bias. However, because the data was collected in the participants’ homes, it was not practically, logistically, or financially feasible to have a second set of blinded research staff whose purpose was solely to collect data. Stopping recruitment before target enrollment numbers were reached likely limited our ability to detect statistically significant differences between treatment arms, especially given the low compliance rates with visits observed in the PATE arm (Thomson JL, under review). A post hoc power and sample size analysis revealed that the actual sample sizes of 33 PAT and 26 PATE participants were sufficient to detect a statistically significant difference of 9.9 points in change in total HEI-2010 scores between treatment arms with 80 % power and a type 1 error rate of 0.05. Given the relatively short duration of the intervention in the gestational period, achieving such a large effect was unlikely. Finally, the high attrition rate may limit generalizability of these study results.

## Conclusion

In conclusion, designing effective interventions that enable women to improve their dietary intake during pregnancy remains a challenge as evidenced by the lack of impact on maternal diet quality observed in the current study. Future interventions designed for rural, low income, minority populations of pregnant women might consider recruiting women earlier in their pregnancy (e.g., first trimester), increasing the treatment dosage, and involving prenatal care providers to ensure reinforcement and consistency in health behavior messaging. Given the importance of optimum maternal nutrition for positive maternal and fetal health outcomes, research efforts need to remain focused on populations at risk for poor pregnancy and birth outcomes, including residents of the LMD region.

## Abbreviations

BMI, body mass index; DGAs, dietary guidelines for Americans; DPP, Diabetes Prevention Program; GM, gestational month; HEI, healthy eating index; InFANT, Infant Feeding and Nutrition Trial; LMD, Lower Mississippi Delta; NDSR, nutrition data system for research; PAT, parents as teachers; PATE, Parents as Teachers enhanced curriculum; US, United States; USDA, United States Department of Agriculture; WIC, Women, infants, and children
